# An innovative automated active compound screening system allows high-throughput optimization of somatic embryogenesis in *Coffea arabica*

**DOI:** 10.1038/s41598-020-57800-6

**Published:** 2020-01-21

**Authors:** Rayan Awada, Dorothée Verdier, Solène Froger, Eric Brulard, Simone de Faria Maraschin, Hervé Etienne, David Breton

**Affiliations:** 1 Nestlé R&D Center Tours, 101 avenue Gustave Eiffel, 37097 Cedex 2 Tours, France; 2 CIRAD, UMR IPME, F-34398 Montpellier, France; 3 UMR IPME, Université de Montpellier, CIRAD, IRD, F-34398 Montpellier, France; 4 Bioalternatives SAS, 1 bis Rue des Plantes, 86160 Gençay, France

**Keywords:** Chemical libraries, Plant regeneration

## Abstract

Somatic embryogenesis (SE) faces many challenges in fulfilling the growing demand for elite materials. A high-throughput approach is required to accelerate the optimization of SE protocols by multiplying experimental conditions within a limited time period. For the first time in plant micropropagation, we have developed a miniaturized and automated screening system to meet high-throughput standards. *Coffea arabica* embryo regeneration, classically achieved in 250-ml Erlenmeyer flasks, was successfully miniaturized in 24-well plates, allowing a volume downscaling factor of 100 and a space saving of 53 cm^2^/well. Cell clusters were ground and filtered to fit the automated pipetting platform, leading to fast, reproducible and uniform cluster distribution (23.0 ± 5.5 cell clusters/well) and successful regeneration (6.5 ± 2.2 embryos/well). Pilot screening of active compounds on SE was carried out. Compounds belonging to the histone deacetylase inhibitor family were tested for embryo regeneration efficiency. Cells treated with 1 µM Trichostatin A showed a marked 3-fold increase in the number of regenerated embryos. When re-tested in 250-ml flasks, the same enhancement was obtained, thereby validating the miniaturized and automated screening method. These results showed that our screening system is reliable and well suited to screening hundreds of compounds, offering unprecedented perspectives in plant micropropagation.

## Introduction

The rapid increase in environmental stresses, especially those associated with climate change, and growing demand from the food and timber industries, have put enormous pressure on trees^1^. Developing new technologies for the clonal propagation, improvement and breeding of trees can help solve these problems^2^. This has been achieved in part using biotechnology methodologies, such as *in vitro* propagation, genetic transformation, and marker-assisted breeding for the gradual genetic improvement of woody plants^3,4^.

For *in vitro* propagation, shoot proliferation methods using adventitious shoots and axillary buds have been widely applied, though research is still largely focused on using somatic embryogenesis (SE) in woody plants^5^. Today, SE is considered as a very promising tool for the rapid and large-scale propagation of elite varieties in many species^5,6^, and is particularly important for those that have a long life cycle and are difficult to propagate by conventional methods^7^.

However, to date, very few examples of commercial SE applications are available. The industrial application of SE in hardwoods remains limited to a few species, such as the hybrid yellow-poplar, cocoa, and coffee^8^. SE technology is still work-in-progress, mainly due to low biological efficiency and high genotypic effects resulting in high production costs^6,9^.

To guarantee the security of the supply chain in response to market demand for elite materials, it is necessary to maximize SE-derived plant production and quality, i.e. to improve SE protocols. Whatever the plant species, a number of common bottlenecks have been noted throughout the main three steps of the current SE process –(1) the induction of embryogenic callus, (2) its proliferation, and (3) its differentiation into embryos. For instance, a strong genotypic effect has often been reported in the first step^10^. Explants of some interesting clones either do not respond to auxin stimulus, or respond by directly producing embryos without passing through a proliferating callus step. For the second step, calli of some clones lose their embryogenic potential during proliferation^11^. For the third step, maximizing embryo yields is a necessity to secure the supply chain and reduce production costs^12^.

Until now, one of the conventional methods for improving SE protocols has consisted in applying active compounds. Adding small (<500 Da) bioactive compounds (nutrients, growth regulators, epigenetic regulators, etc.) to the culture medium that have a potential effect on SE targets, and are capable of modulating the physiological state of cells, is a possible way of maximizing embryo production and quality, and of removing genetic or stress-induced recalcitrance^13^. The ability to use small compounds to improve tissue culture responses eliminates the need to create and market transgenic plants^14^. This is important in the food sector, where consumers are reluctant to consume transgenic products.

However, testing the effect of new compounds on SE efficiency under standard conditions is generally a laborious, intensive and tricky operation, because it is time-consuming, space-demanding and costly. To date, the literature on SE in several species has shown that only a limited number of compounds have been chosen to be tested, on the basis of prior hypotheses (targeted selection)^15–17^ and when tested, only a very limited range of concentrations was chosen. The above inconveniences also hampered the possibility of testing these compounds in combinations. The resulting low-throughput research strategy has led to overall slow technical progress on SE protocols in recent years.

At the same time, high-throughput strategies are gaining increasing attention, particularly in the fields of biofuel production and pharmaceuticals^18,19^. These strategies consist in testing a large number of compounds at different concentrations (untargeted selection) in a miniaturized and automated system, to systematically screen for novel modifiers of a biological phenomenon. They combine large-scale chemistry and biology data, along with bioinformatics, which is required for data mining^20^. The effectiveness of these strategies is enhanced by the fact that most active compounds are small molecules that modulate target proteins and/or pathways of a determinate biological process^21^. In the last decade, several collections of bioactive compounds became available for the research community, enabling the screening of hundreds of libraries for novel activities^22^. Nevertheless, to our knowledge, this kind of approach has never been applied to optimizing culture media for micropropagation, particularly for SE.

In plants and mammals, epigenetic regulation and histone modifications occur widely during cellular differentiation and development^23,24^. Nic-Can *et al*.^25^ showed that genes related to SE, such as *LEAFY COTYLEDON1, BABY BOOM1* and *WUSCHEL-RELATED HOMEOBOX 4*, are epigenetically regulated in *Coffea canephora*. Boutilier *et al*.^14^ showed that the switch to haploid embryogenesis is controlled by the activity of histone deacetylases (HDAC). Blocking HDAC activity with HDAC inhibitors (HDACi) in *Brassica napus*, *Brassica rapa*, *Arabidopsis thaliana* and *Capsicum annuum* male gametophytes resulted in a large increase in the proportion of cells undergoing embryogenic growth^14^.

Coffee (C. *arabica* & C. *canephora* cv Robusta) has a great economic impact in many producing countries, especially in South America^26^. Today, coffee SE is one of the most advanced technologies. Research on SE by two leading groups - the CIRAD/ECOM alliance and Nestlé - has led over the last 13 to 15 years to the successful industrialization and commercial application of SE for the two cultivated species, through the large-scale dissemination of Arabica F1 hybrids and Robusta clones^8^. Even though the processes established in the 2 species display good biological efficiency, with low genotypic effects^27,28^ and controlled somaclonal variation^11,29^, SE production costs remain high and still cannot compete with the production costs of conventional seedlings or cuttings^30^.

While the micropropagation of coffee *in vitro* plantlets can today reach 2–3 M SE-derived plants per year, SE faces many challenges in fulfilling growing market demand for elite coffee varieties, estimated at around 50–100 M plants per year^8^. This market demand for more resilient varieties comes from the recent loss of leaf rust resistance in all the conventional cultivated line varieties for Arabica, and the increasing impact of the harmful effects of climate change. For Robusta, there is also an urgent need to replace unselected clones with improved ones, particularly in Vietnam – the world’s main coffee producer – where aging coffee orchards need to be totally renewed^8^.

As for other woody species, optimizing coffee SE protocols is a long and laborious process. Yet, increasing production efficiency *in vitro* would make SE technology more cost-effective, hence the interest in finding new active compounds able to increase embryogenic callus formation and embryo yield. A high-throughput approach might be a solution, given the possibility of multiplying experimental conditions in a limited time period.

We focus in this paper on one of the main three SE steps in *C. arabica*: embryo regeneration from embryogenic cell clusters. This SE step is classically achieved in 250-ml Erlenmeyer flasks. Here, we describe how we miniaturized this step in 24-well plates and made it compatible with an automated pipetting platform. Cell cluster miniaturization was also required. Once miniaturization and automation were successful, we carried out pilot screening using compounds belonging to the HDACi family, for their potential role as active compounds to increase embryo yield. Lastly, treatments showing a significant positive effect on embryo yield were tested again under standard *in vitro* conditions to confirm treatment efficiency and validate the automated and miniaturized screening method.

## Results

### Grinding and filtration of embryogenic tissues to overcome the pipetting constraint

**Figure 1:**
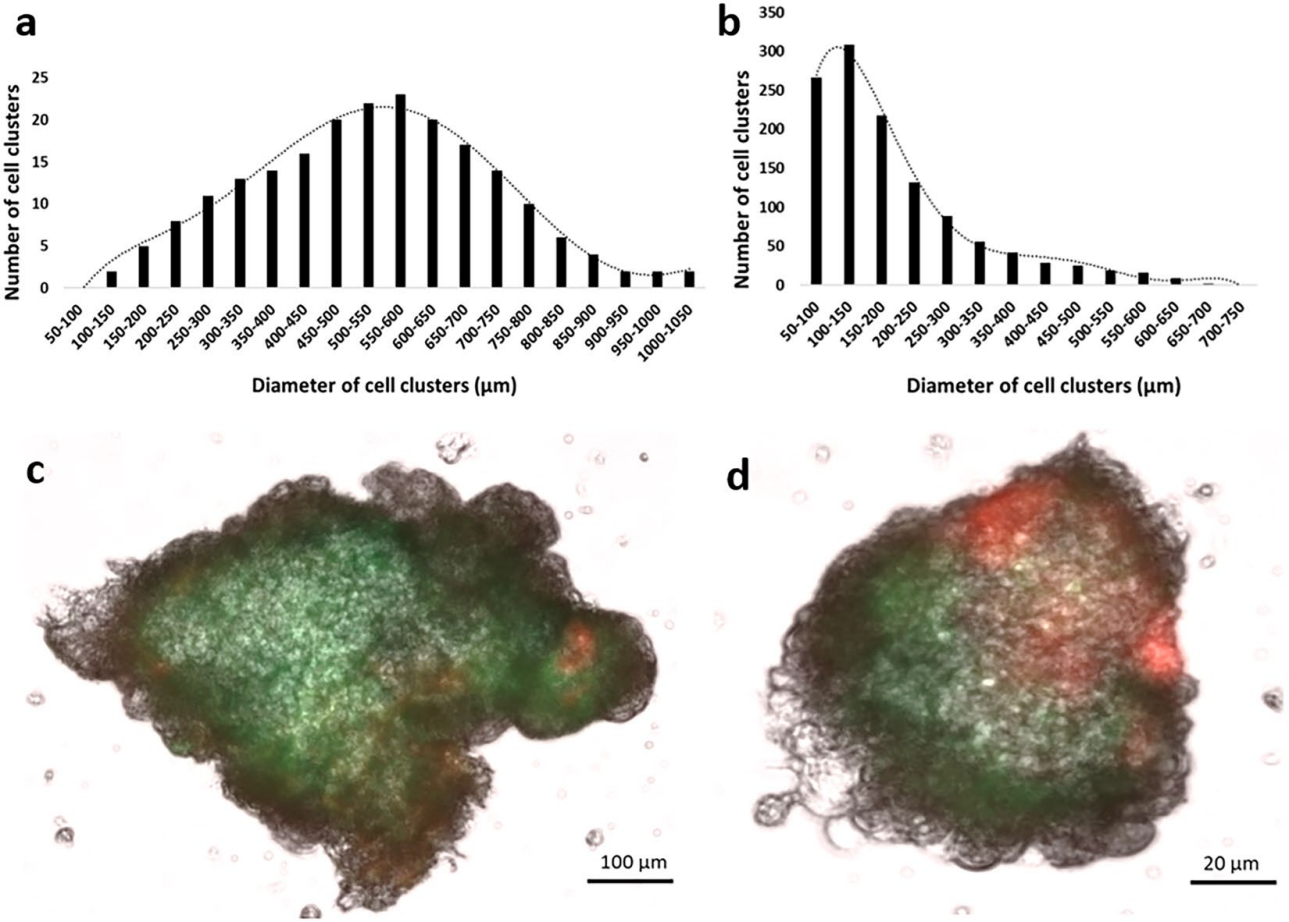
Successful miniaturization of Arabica embryogenic cell clusters. **(a)** Profiling of cell clusters according to their diameter before grinding and filtration. Data of 210 cell clusters were plotted. The black curves show that the plotted values are normally distributed. **(b)** Profiling of cell clusters according to their diameter after grinding and filtration at 700 µm. Data of 1210 cell clusters were plotted. Viability of cell clusters before **(c)** and after grinding **(d)** following double staining with FDA and PI. FDA fluoresces in green in living cells and PI fluoresces in red in dead cells.

Preliminary profiling of long-term cultured Arabica embryogenic cell clusters showed that their diameter fell within the range of 100–1000 µm and followed a statistical normal distribution, with a maximum peak around 500–600 µm (Fig. 1a). For automation purposes, uniformity and reproducibility are required. Hence, in order for the cell clusters to fit the pipetting tips, filtration at 700 µm was needed to exclude a diameter greater than 700 µm, which amounted to approximately 40% of the whole cell suspension. To benefit from all the available embryogenic material at our disposal, we decided to add a grinding step to reduce and homogenize cell cluster diameter. Figure 1b shows the effect of grinding on the particle diameter distribution. Cell cluster diameters maintained their statistical normal distribution, but with a shift of the maximum peak towards the range of 100–150 µm. Viability tests (Fig. 1c,d) showed similar staining profiles between ground and intact cell clusters, with a prevailing green light emission indicating that cells had high viability. Some peripheral cells emitted red light, indicating their dead status in both ground and intact clusters, though the red emission intensity was more noticeable in ground cells, due to the effect of the grinding blades.

**Figure 2:**
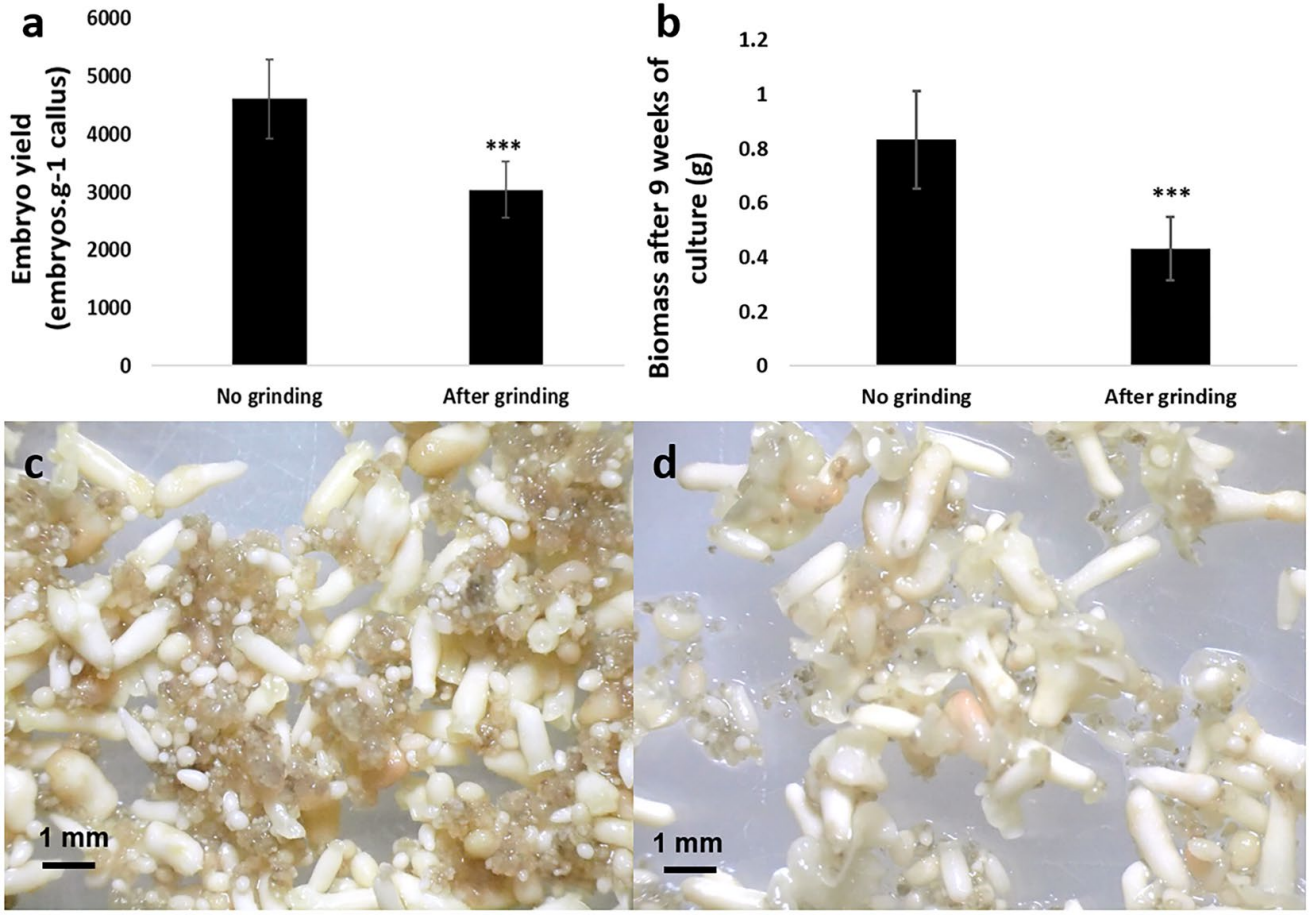
Assessing the effect of cell cluster grinding on embryo regeneration in Erlenmeyer flasks. (**a**) Embryo yield (embryos. g^−1^ callus) obtained after 9 weeks of embryo regeneration. **(b)** Biomass (g) obtained after 9 weeks of embryo regeneration. Bars show the mean of triplicate samples (n = 6 flasks/condition/replicate) and error bars represent the SD. Differences between both groups were analysed with t-tests (***P < 0.001). **(c)** Appearance of torpedo-shaped embryos (white structures) obtained after 9 weeks of embryo differentiation from intact cell clusters. Remaining embryogenic clusters can still be observed (brown structures). **(d)** Appearance of torpedo-shaped embryos regenerated after 9 weeks of differentiation from ground cell clusters. Better synchronization of embryo development is observed.

The regeneration capacity of ground material had first to be tested under standard *in vitro* culture conditions (Erlenmeyer flasks) (Fig. 2). After 9 weeks of culturing, an average of 3,039 ± 485 normal torpedo-shaped embryos were obtained per gram of inoculated callus (embryos. g^−1^ callus) representing a biomass of 0.43 ± 0.12 g (n = 6 flasks/condition/replicate). These values were significantly lower compared to those obtained with intact cell clusters, i.e. 4,611 ± 685 embryos. g^−1^ callus and a biomass of 0.83 ± 0.18 g (***P < 0.001) (Fig. 2a,b). However, it should be noted that better synchronization of embryo development was observed. Embryos generated from ground cell clusters were morphologically more uniform, since very few proliferating clusters remained, limiting spatial competition (Fig. 2c,d).

### Achieving uniform cell cluster distribution after automated pipetting in miniaturized culture containers

**Figure 3:**
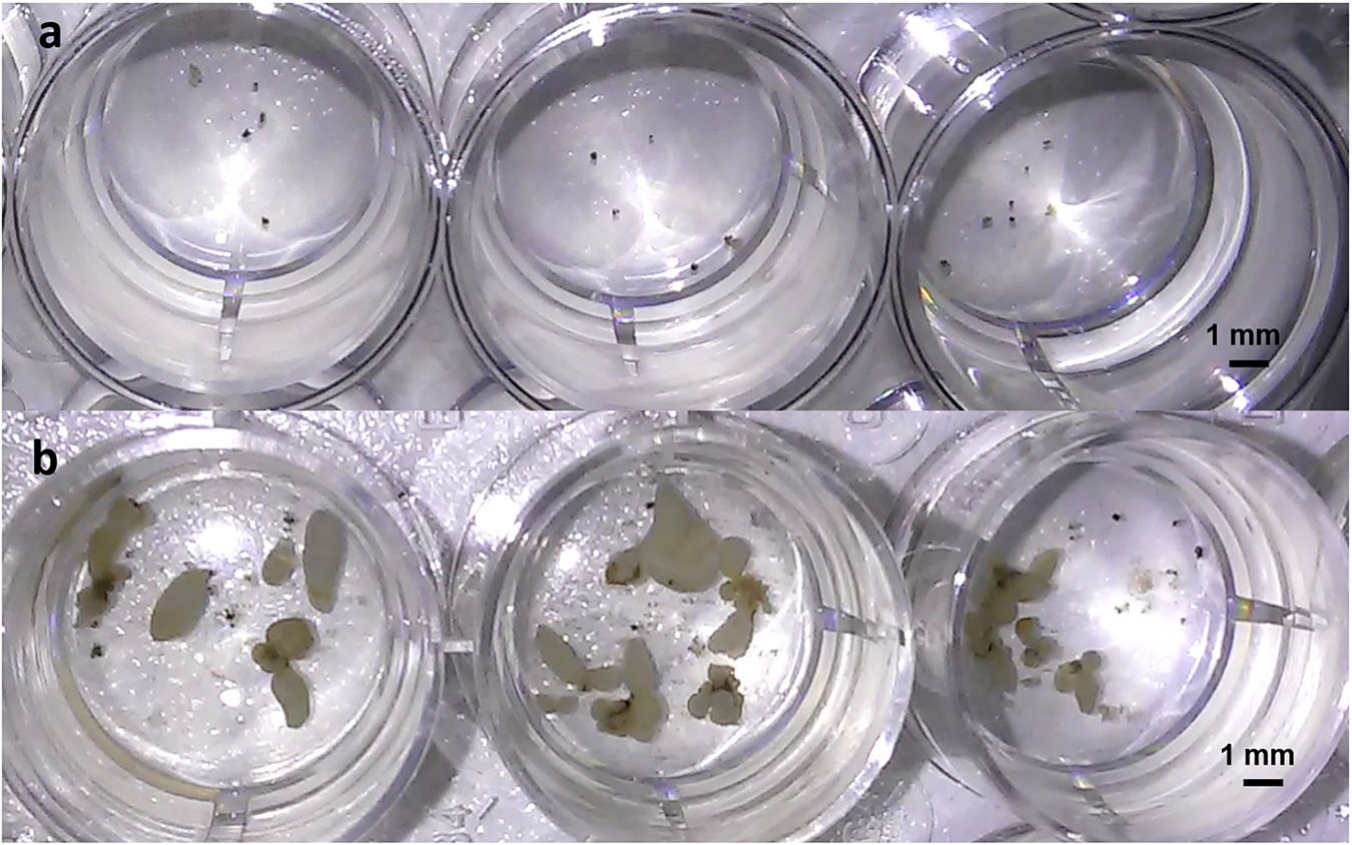
Successful uniform distribution of Arabica cell clusters resulting in a uniform number of regenerated embryos. Programming the automated pipetting platform resulted in uniform distribution of cell clusters between wells at the beginning of the regeneration step as well as a uniform number of embryos regenerated after 9 weeks. **(a)** Appearance of inoculated cell clusters in a 24-well plate after distribution using the automated pipetting platform after cell cluster grinding and filtration. **(b)** Appearance of embryos regenerated from ground clusters in a “24-well plate” miniaturized culture system.

In order to obtain uniform distribution of cell clusters at the start of culturing, two parameters were quantified to verify uniformity of distribution: the number of cell clusters in each well, and the total area they occupied. The values of 4 independent replicates (n = 24 wells/replicate) were uniform (P > 0.05) and similarly distributed between plates (1, 2, 3, 4) and between rows (A, B, C, D), having an average of 23.0 ± 5.6 clusters/well and occupying an area of 1.39 ± 0.12 mm^2^/well (Table 1, Fig. 3a).

**Table 1:** Assessing the uniformity and reproducibility of automated distribution of Arabica cell clusters in 24-well plates.

Parameters	Assessment of distribution uniformity									
	Between plates				Between rows				Between central and peripheral wells	
	Plate 1	Plate 2	Plate 3	Plate 4	Row A	Row B	Row C	Row D	Central wells	Peripheral wells
Number of cell clusters per well	22.1 ± 5.7*a*	24.4 ± 6.0*a*	22.0 ± 5.5*a*	23.3 ± 5.0*a*	21.1 ± 4.7*a*	24.5 ± 5.4*a*	22.5 ± 5.0*a*	23.8 ± 6.6*a*	23.9 ± 5.6*a*	22.5 ± 5.4*a*
Total area occupied (mm²)	1.40 ± 0.10*a*	1.41 ± 0.12*a*	1.37 ± 0.13*a*	1.38 ± 0.12*a*	1.39 ± 0.11*a*	1.38 ± 0.10*a*	1.41 ± 0.12*a*	1.38 ± 0.15*a*	1.39 ± 0.11*a*	1.39 ± 0.13*a*
Embryo yield per well	6.9 ± 2.5*a*	6.7 ± 2.4*a*	6.2 ± 1.7*a*	6.3 ± 2.2*a*	7.0 ± 2.9*a*	6.5 ± 1.8*a*	6.2 ± 2.2*a*	6.4 ± 1.8*a*	6.3 ± 2.0*a*	6.6 ± 2.3*a*

Uniformity of distribution was revealed between plates, between rows, and between central and peripheral wells. Three parameters were quantified: the number of clusters per well and the total area they occupied (mm²) at the beginning of the regeneration step, and the number of regenerated embryos per well after 9 weeks. Values represent the mean ± SD of 4 independent replicates (n = 24 wells/replicate). For each parameter, differences between plates and between rows were analysed with a one-way ANOVA test, while a t-test was used to analyse differences between central and peripheral wells. The letter “a” shows no significant difference between groups (P > 0.05).

The preliminary results showed medium evaporation in peripheral wells (loss of 40% of total volume after 4 weeks compared to central wells) causing non-reproducible embryo regeneration (data not shown). Parafilm-sealed plates were then placed in a culture chamber with 90% RH, which drastically limited the evaporation rate to less than 2% in all wells, and led to uniformity between tested replicates (P > 0.05). Similar distribution of biomass parameters was obtained between peripheral wells (22.5 ± 5.39 cell clusters/well; area occupied 1.39 ± 0.13 mm^2^) and central wells (23.9 ± 5.63 clusters/well; area occupied 1.39 ± 0.11 mm^2^) (Table 1).

### Maintenance of regeneration capacity and embryo yield reproducibility in a miniaturized environment

**Figure 4:**
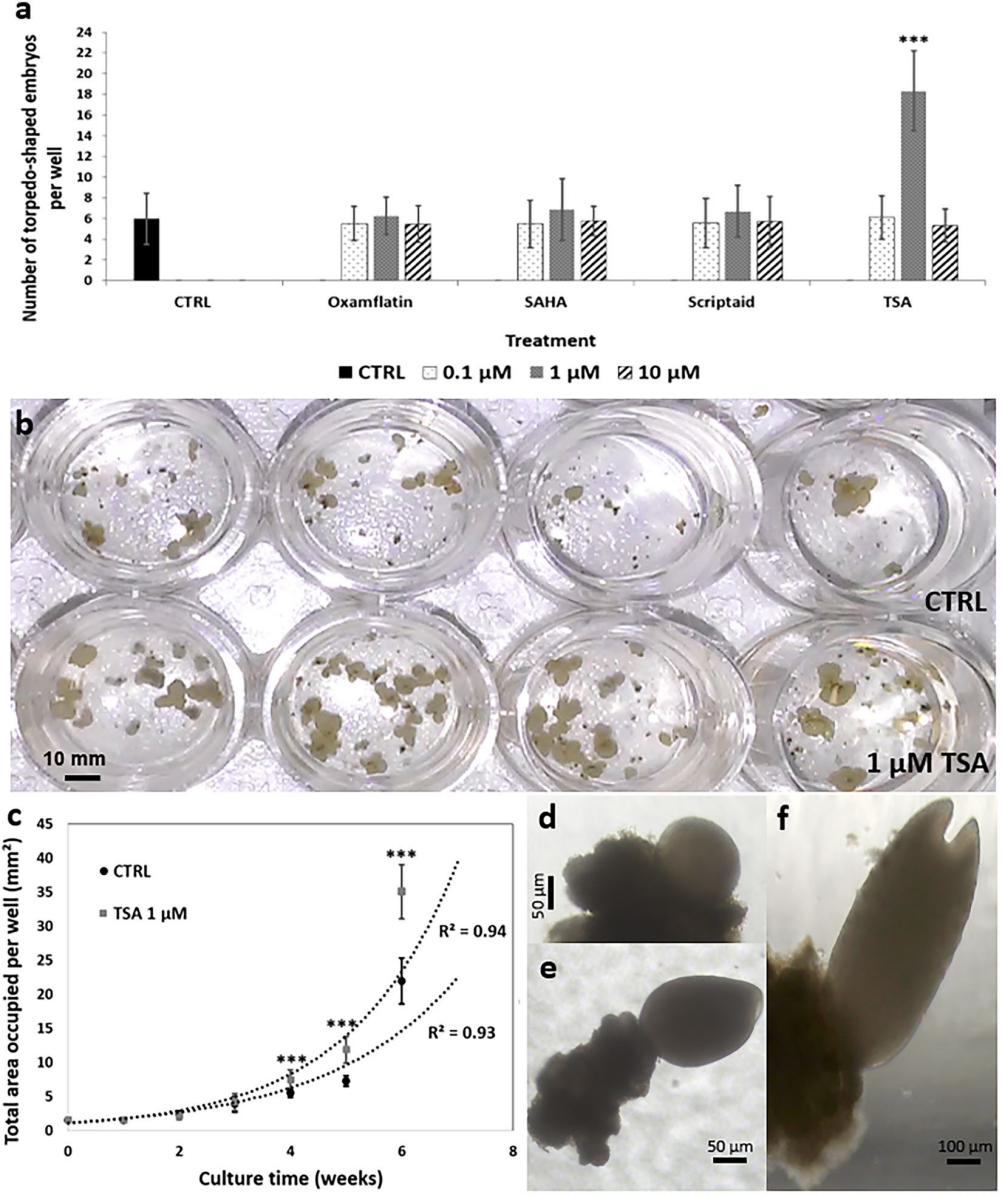
Successful pilot screening of active compounds affecting SE in the automated and miniaturized system. Four compounds belonging to the HDACi family (Oxamflatin, Scriptaid, SAHA and TSA) were tested in 24-well plates at three different concentrations (0.1, 1 and 10 µM), as potential active compounds affecting *C. arabica* embryo differentiation. Cell clusters were exposed for 24 h to treatment before washing. **(a)** Embryo regeneration was scored after 9 weeks. The “CTRL” group was untreated (control). Bars show the mean of triplicate samples (n = 18 wells/HDACi/concentration/replicate) and error bars represent the SD. Differences between treated clusters and the control were analysed with a one-way ANOVA test (***P < 0.001). **(b)** Picture showing the increased embryo yield in the wells treated with 1 µM TSA (bottom) vs control wells (top). **(c)** The total area occupied by cell clusters was monitored during the embryo regeneration period. Plotted values are the mean of triplicate samples (n = 18 wells/treatment/replicate) and error bars represent the SD. Differences between clusters treated with 1 µM TSA and the control were analysed with a t-test (***P < *0.001*). Cell cluster growth followed an exponential trendline (R² = 0.93) up to the sixth week. **(d–f)** Pictures of an emerged globular-shaped, heart-shaped and torpedo-shaped embryo after 6, 7 and 8 weeks under embryo regeneration conditions, respectively.

The efficiency of embryogenic cell cluster conversion to embryos was validated in the 24-well plate culture system. The regenerated embryos were normally shaped, with a conversion rate of 6.5 ± 2.1 torpedo-shaped embryos/well after 9 weeks (Table 1, Fig. 3b). The values of 4 independent replicates (n = 24 wells/replicate) followed a statistical normal distribution. High uniformity in embryo yield rates was found between plates, rows and peripheral and central wells (P > 0.05), confirming the very limited impact of automated pipetting on cluster distribution, whatever the well position. However, it should be noted that expressing this embryo yield in wells by gram of inoculated callus resulted in a mean of 13,000 ± 4,200 embryos, which was higher than the embryo yield obtained in a flask culture system from ground or intact cell clusters, i.e. 3,039 ± 485 embryos and 4,611 ± 685 embryos, respectively. However, for the same inoculated cell cluster density, a delay in embryo development was found in the miniaturized system, where the first globular-shaped embryos emerged after 6 weeks (Fig. 4d–f), as opposed to 4 weeks in Erlenmeyer flasks.

### Successful pilot screening for the effect of active compounds on somatic embryogenesis

After satisfactory implementation of a miniaturized and automated embryo regeneration assay, pilot screening was carried out by testing histone deacetylase inhibitor (HDACi) treatments. Four compounds belonging to the HDACi family – Oxamflatin, SAHA, Scriptaid and Trichostatin A – were tested at 3 different concentrations (0.1, 1 and 10 µM) as potential active compounds affecting *C. arabica* embryo differentiation. The regenerated embryo numbers of 3 independent replicates (n = 18 wells/HDACi/concentration/replicate) were uniform between plates and followed a statistical normal distribution between culture wells. Only clusters treated with 1 µM TSA showed a significant increase in the number of torpedo-shaped embryos obtained after 9 weeks (18.3 ± 3.9 embryos/well vs 6.0 ± 2.5 for untreated control, ***P < 0.001) (Fig. 4a,b) and the appearance of the regenerated torpedo-shaped embryos was comparable between clusters treated or not with TSA (Fig. 4b). The area occupied by cell clusters in a well was recorded over a 6-week culture period up to the emergence of globular-shaped embryos (Fig. 4c,d). Comparing the resulting curves established with cell clusters treated with 1 µM TSA and untreated clusters showed that cluster growth started after 3 weeks and exponentially increased up to the sixth week of culturing (R² = 0.93), when the first globular-shaped embryos emerged (Fig. 4d). From 4 weeks under embryo regeneration conditions, the growth of cell clusters treated with 1 µM TSA was significantly better compared to the untreated control (total area of 35.0 ± 3.9 mm^2^ vs 21.9 ± 3.4 mm^2^ after 6 weeks, ***P < 0.001), explaining the larger number of regenerated embryos obtained after 9 weeks (Fig. 4a).

**Figure 5:**
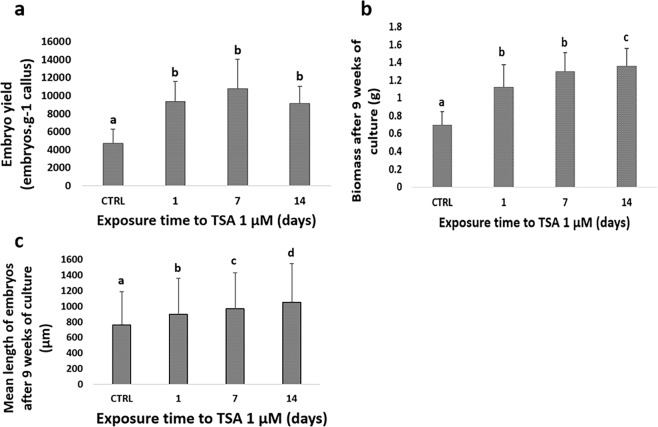
Validation in Erlenmeyer flasks of active compounds highlighted in the pilot screening. Treatment with 1 µM TSA was re-tested in standard 250-ml Erlenmeyer flasks with an exposure time varying between 1, 7 and 14 days. **(a)** Embryo yield (embryos. g^−1^ callus) obtained after 9 weeks. **(b)** Biomass (g) obtained after 9 weeks. **(c)** Embryo length. Bars show the mean of triplicate samples (n = 6 flasks/exposure time/replicate for (**a**,**b**) and n = 100 embryos/exposure time/replicate for (**c**)) and error bars represent SD. The “CTRL” group was untreated (control). Differences between treated clusters and the control were analysed with a one-way ANOVA test for (**a,b**), while a Kruskal-Wallis test was used for (**c**) followed by a Dunn test as a post hoc. Means with different letters are significantly different (*P* < 0.001).

To validate the screening carried out in the automated and miniaturized system, the best treatment obtained with 1 µM TSA, with an exposure time of 24 hours, was re-tested under standard *in vitro* conditions, i.e. 250-ml Erlenmeyer flasks. The TSA treatment enhanced embryo regeneration in the same way compared to the untreated embryogenic clusters (9,325 ± 2,240 embryos. g^−1^ callus vs 4,673 ± 1,614 embryos. g^−1^ callus, respectively; P < 0.001; Fig. 5a). The beneficial effects of TSA were also visible through the increased overall biomass (1.12 ± 0.26 g vs 0.69 ± 0.15 g, P < 0.001; Fig. 5b), and the increased length of the treated torpedo-shaped embryos (896 ± 463 µm vs 760 ± 429 µm, respectively, P < 0.001; Fig. 5c). Extended exposure to 1 µM TSA, which was also tested (7, 14 d), did not significantly improve the embryo regeneration yield compared to an exposure time of 24 h, but biomass and torpedo mean size significantly increased with an exposure of 14 d to 1 µM TSA.

### Treating cell clusters with TSA does not affect plant regeneration

**Figure 6:**
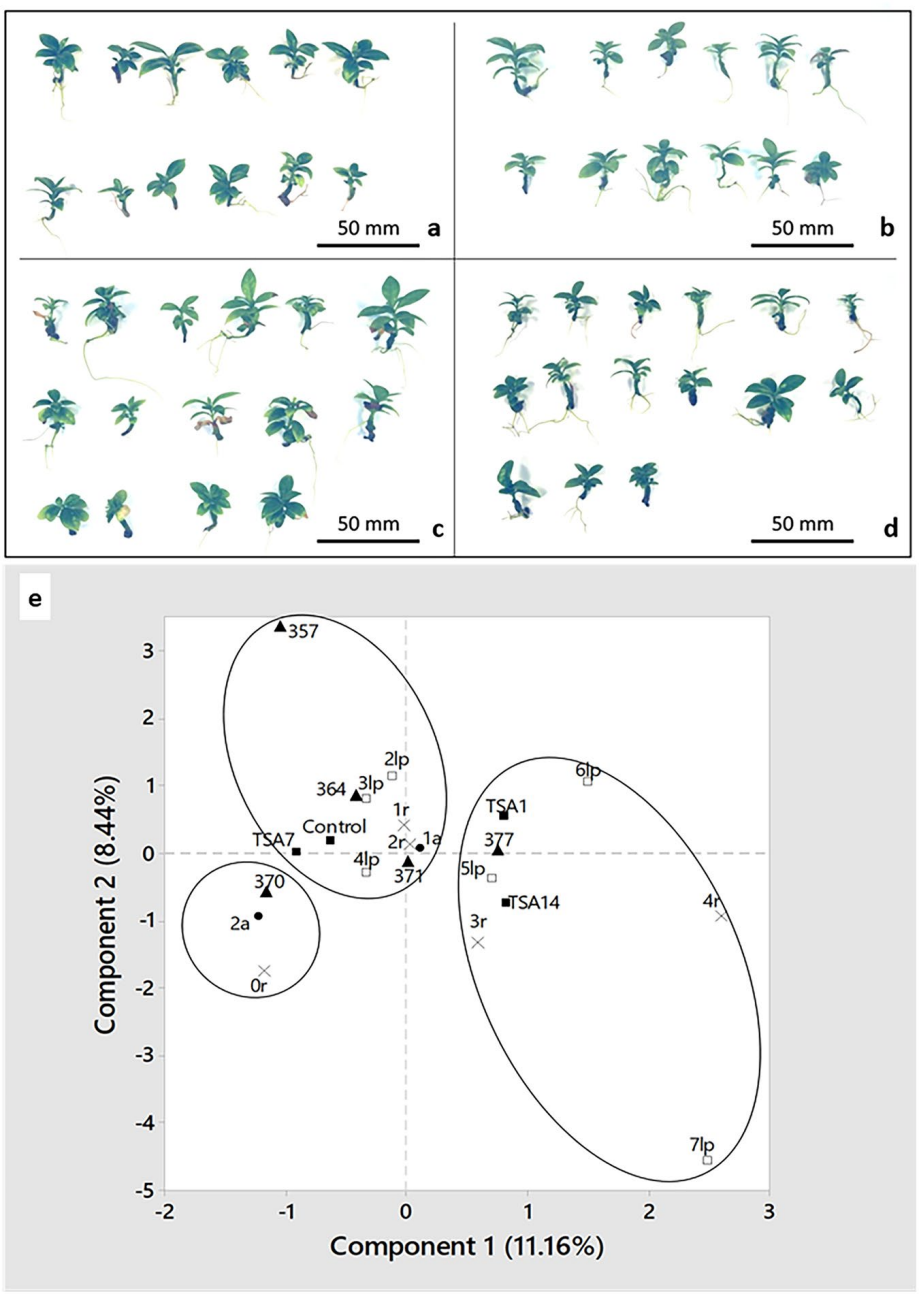
*In vitro* plant phenotypic characterization following 1 µM TSA treatment during the coffee SE process. (**a**) Plants regenerated from untreated embryogenic callus. **(b–d)** Plants regenerated from TSA-treated embryogenic clusters for 1, 7 and 14 days, respectively. **(e)** Graphic representation of a Multiple Component Analysis (MCA) performed on plant phenotypic variables. Data from 157 plants were plotted according to the first 2 axes of the MCA. The number of pairs of leaves are represented by white squares, the number of roots by crosses, the number of stems by black spots, the leaf colour index by triangles and the *in vitro* treatment prior to regeneration by black squares.

The phenotypic data recorded on 160 young plants ready for greenhouse transfer did not reveal any significant differences between plants regenerated from cell clusters treated, or not, with TSA. An ANOVA did not indicate any treatment effect on either main root or stem lengths (data not shown). All plants displayed orthotropic development, with uniform pigmentation and leaf shape (Fig. 6a). A Multiple Component Analysis (MCA) performed on the scores obtained for the main phenotypic categorical variables (number of pairs of leaves, number of roots, number of stems, leaf colour index, Table 2) provided 19 components reaching 3.8 inertia (data not shown). However, the first two axes accounted for only 18.9% of total inertia. Plotting categorical data according to axes 1 and 2 revealed a strong contribution to global variability for 2 plants scored “376” and one scored “382” for their colour index (Supplementary Data). As a result, no distinction could be detected between TSA treatments so we decided to discard these data and run another MCA. This time, 17 components were detected with a total level of 3.4 inertia (data not shown). The contribution of the first two axes to this inertia reached 19.6%. This time, a graphical analysis revealed 2 main groups of categorical data and a smaller third one including atypical, double stem and unrooted plants (Fig. 6e). This group did not include TSA treatment or control conditions, indicating a lack of correlation between variables. Considering the upper left of the graph, plants from untreated tissues and tissues treated for 7 days with TSA correlated with plants bearing 2 to 4 pairs of leaves, 1 to 2 roots and various leaf colour indexes (357, 364, 371). Conversely, on the right of the graph, plants arising from cell clusters treated for 1 and 14 days correlated with well-developed plants: 5 to 7 pairs of leaves, 3 to 4 roots, and a stable colour index (377). Thus, treating cell clusters with 1 µM TSA did not show any significant deleterious effect on regenerated plants.

**Table 2:** Phenotypical distribution of the 160 acclimated plants as a function of the TSA treatments *in vitro*. Each plant was individually scored for their leaf pairs (lp) number, roots (r) number, stems (a) number and colour index. Treatments consisted in growing *in vitro* embryogenic clusters in contact with 1 µM TSA for 0 (control), 1, 7 and 14 days.

Phenotypic scores								
**Leaves**	**2lp**	**3lp**	**4lp**	**5lp**	**6lp**	**7lp**		**Total**
Control		12	18	10				40
TSA1		13	14	11	2			40
TSA7		6	15	16	2	1		40
TSA14	1	17	16	6				40
Total	1	48	63	43	4	1		160
**Roots**	**0r**	**1r**	**2r**	**3r**	**4r**			**Total**
Control	5	20	13	2				40
TSA1	1	20	15	3	1			40
TSA7	3	17	14	5	1			40
TSA14	3	19	14	4				40
Total	12	76	56	14	2			160
**Stems**	**1a**	**2a**						**Total**
Control	36	4						40
TSA1	38	2						40
TSA7	37	3						40
TSA14	36	4						40
Total	147	13						160
**Colour index**	**357**	**364**	**370**	**371**	**376**	**377**	**382**	**Total**
Control	1	11	13	6	1	8		40
TSA1		2		5		33		40
TSA7		3	4	4	1	27	1	40
TSA14		9	20	4		7		40
Total	1	25	37	19	2	75	1	160

## Discussion

We proved in this study that a major step of SE processes, i.e. embryo regeneration from embryogenic cell clusters, can be successfully miniaturized and automated allowing easy, quick, cheap and reliable testing of numerous compounds at varied concentrations. Switching from conventional 250-ml flask culture containers filled with 100 ml of culture medium to 24-well plates with 1 ml of available nutritive medium allowed a miniaturization factor of 100. With this strong downscaling, consideration could be given to testing the effectiveness of a much larger number of molecules at different concentrations, while reducing the space required (2.5 cm^2^ required per well compared to 55.4 cm^2^ required per flask and the possibility of stacking multi-well plates). In addition, automation led to a considerable gain in speed and reduced manpower, where only 1 working full-time employee (FTE) was needed to supervise the pipetting platform. For instance, the pilot screening carried out using HDACi treatments required 36 plates, 864 wells, i.e. 864 independent culture environments. The same experiment in flasks was simply unmanageable, as 1 FTE can only inoculate 100 flasks a day, not to mention the space required, whereas inoculation of several hundred wells can be done within hours. Many authors have also shown the importance of miniaturization and automation in animal or plant cell screening systems^31–33^. For instance, Rodriguez-Furlan *et al*.^33^ also showed that the 24-well microplate format used to screen for compounds modulating *A. thaliana* seedling growth required a minimum of sample handling and allowed automatic acquisition of quantitative data and rapid monitoring of morphological traits (leaf and root growth). This container format, along with automation, allowed the screening of 10,000 structurally diverse compounds in a limited time period.

While animal cell screening systems are mainly derived from drug discovery programmes and cancer research^18,34^, plant cell screening systems were only recently used to study compound effects on growth and development. To date, in plants, only *A. thaliana* seedlings have been used in such studies, because of their small size, allowing development in multi-well containers^33,35^. However, automated screening systems for active compounds have never been used in plant propagation procedures. Our system provided the first evidence in plants that miniaturized containers do not hamper embryogenic cell cluster growth and differentiation, and thus enable screening for the effectiveness of compounds in micropropagation, under real conditions and on economically important crops.

Our results showed that even after miniaturizing the embryo regeneration step, mechanisms occurring during conventional-scale SE were preserved, as reliable production of well-shaped embryos was achieved. SE miniaturization did not engender any developmental heterogeneity. However, the type of grinding chosen had significant effects by lowering embryo yields in flasks. Since this biological efficiency was reproducible during this step, we did not consider this difference to be a drawback, because the main objective of this miniaturized system was not large-scale embryo production, or to study molecular mechanisms underlying embryogenesis, but to use it on SE as a tool to screen for active compounds, with a view to speeding up protocol optimization.

A difference in embryo development timing was also noted between the Erlenmeyer flask and 24-well plate culture systems, which could be explained by obviously different physico-chemical environments. While rotary shaking was set to the same speed for both systems (120 rpm), it showed different effects, mainly with fast spatial movement of the nutritive medium in flasks, compared to slow movement in wells, due to their smaller diameter. Such lower shaking is known to affect medium O_2_/CO_2_ uptake^36,37^ and probably explained the differences between the 2 culture systems.

Pilot screening was carried out as proof-of-concept to show that the miniaturized automated culture system we developed was efficient for testing the effect of putatively active compounds on SE, and was reliable and reproducible. Having uniformity of cell cluster distribution is essential before initiating regeneration, to avoid false positives and negatives when screening for active compounds. The use of an automated scanning microscope enabled a considerable gain in speed and quality in terms of image acquisition and analysis. Proof-of-concept was achieved by proving the stimulation of coffee SE by TSA. A 1 µM TSA supply promoted the proliferation of embryogenic cells and led to a 3-fold increase in embryo yield. When re-tested under standard conditions, treatment with 1 µM TSA confirmed its efficiency, hence validating the whole screening system pipeline. Other authors also showed positive effects of TSA on embryogenic structure formation. Li *et al*.^38^ showed that 0.5 µM TSA promoted totipotency in male gametophytes of *B. napus* and *A. thaliana*. Male gametophytes, which are rather recalcitrant in haploid embryo development in cultures, formed embryogenic cell clusters after TSA treatment. In agreement with these authors, we hypothesize that the 0.1 µM TSA treatment used in our study was at suboptimal concentration to have any observable effect on SE expression while a 10 µM TSA treatment was toxic to cell clusters preventing embryogenesis to occur. Li *et al*.^38^ associated the TSA mode of action with hyper-acetylation of histones H3 and H4 to block histone deacetylase (HDAC) activity. Wójcikowska *et al*.^39^ recently showed that treatment of *A. thaliana* explants with TSA induces SE without the exogenous application of auxin and, thus, the TSA mode of action is related to the activation of auxin-responsive transcription factor genes. In conifers, TSA caused the de-repression of *LEAFY COTYLEDON* genes and stimulated the initiation of SE from cotyledonary embryos in *Picea abies*^40^ and in *Pinus Sylvestris*^41^, but inhibited embryo germination. For *C. arabica*, 1 µM TSA did not have any significant deleterious effect on plants regenerated from treated cell clusters, and thus can now be considered as an active compound for optimizing SE protocols. As increasing the time of exposure to 1 µM TSA did not differ in embryo yield, we put forward the hypothesis that the mechanism by which TSA triggers its response, i.e. blocking histone deacetylase (HDAC) activity, had already occurred on day 1.

This screening system proof-of-concept is a key step towards high-throughput active compound screening platforms. The next step will be to scale-up this technology by screening hundreds of libraries on robotic platforms. Such platforms are widely used in drug discovery programmes^18,36^. They combine robotics, data processing and control software, liquid handling devices, and sensitive detectors to quickly conduct thousands of tests^42^, allowing high-throughput screening (HTS) for active compounds able to modulate a particular molecular pathway. Current generations of platforms are so robust and user-friendly^43^ that HTS can be used to investigate new areas in biology, such as micropropagation. For coffee SE, achieving the intended change of scale, i.e. HTS of hundreds of libraries to optimize SE protocols, still requires three important upgrades to our screening system: (1) new equipment, e.g. high-throughput pipetting platforms; the platforms described above are coupled with a robotic arm and enable full automation of the screening process; (2) new laboratory strategies, e.g. preparation and maintenance of large amounts of embryogenic tissue; (3) new data acquisition strategies; as embryo development occurs in all 3 dimensions, embryo quantification is still difficult to analyse precisely with an automated 2-dimensional scanning microscope. To that end, early SE-markers would be very helpful.

Further high-throughput experiments will require a statistical methodology called “design of experiments”, which helps experimenters to predict how input variables (e.g. active compounds and nutritive compounds) interact to create output responses (e.g. embryo yield) in a process or system (e.g. regeneration process)^44^. Full factorial and fractional factorial experiments are among the most useful multi-factor experiments for scientific investigations^19,45^, in contradiction with a one factor-at-a-time approach used until now to optimize culture media in micropropagation, which requires many test runs and fails to consider any possible interaction between factors.

Thanks to masses of information obtained from “-omics” technologies making it possible to describe, in a global way, the pathways involved in the success of the key SE steps^6^, high-throughput screening can already be piloted in a rational way by selecting libraries able to alter target-molecules playing key roles within these pathways. In many cultivated species, including coffee, sequences of genomes, transcriptomes, proteomes and metabolomes are now available^46–49^. This strategy combining these 2 approaches – “-omics” technologies and robotic screening of active compounds – is currently being implemented by our teams on both cultivated species, *C. arabica* and *C. canephora*, in order to speed up the optimization of SE cloning processes and the dissemination of improved varieties.

Our focus was turned to the SE differentiation phase, where research tends to maximize embryo yields, but other key phases of the coffee SE process could also be easily optimized as a result of the “automated screening of active compound” method, such as the explant-to-callus phase, or the proliferation phase.

These results clearly demonstrate the feasibility, in plants, of automated screening for active compounds in miniaturized SE processes. This proof-of-concept represents a key step towards high-throughput optimization of cell culture media in plant micropropagation. This new method is particularly adapted to SE processes carried out in liquid media and, more widely, to all cell culture-based propagation processes.

## Material and Methods

### Plant material and experimental design to set up a miniaturized and automated screening platform

**Figure 7:**
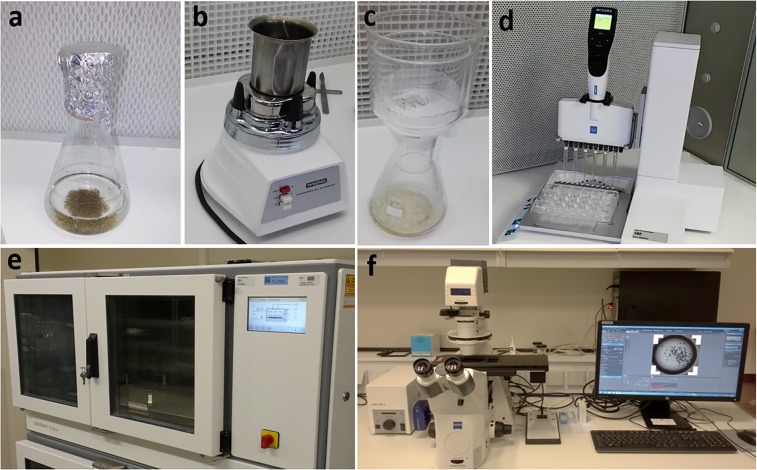
The pipeline established to screen for active compounds affecting coffee SE by miniaturizing and automating the embryo regeneration step. (**a**) Embryogenic cell clusters in M ‘proliferation’ medium. Cell clusters had a heterogeneous diameter range (50–1000 µm). **(b)** Grinding of cell clusters for 30 sec at 18,000 rpm in DIF ‘regeneration’ medium. **(c)** Cell cluster filtration on a 700-µm diameter filter to obtain a uniform diameter range (50–250 µm). **(d)** Automated distribution of ground cell clusters in miniaturized culture systems (24-well plates). The same automated platform was used to screen for potential active compounds affecting SE. **(e)** Plates were placed in the dark in incubation shakers (90% RH 25 °C and 100 rpm) for 9 weeks up to embryo regeneration. **(f)** An automated inverted plate-reading microscope coupled with a digital camera and computer-assisted image analysis were used to count cell clusters, measure the total area they occupied in a well and monitor embryo development.

Embryogenic cell clusters of *C. arabica* cv. GPFA116 were initiated from leaf explants obtained from 1-year-old plants obtained by SE and grown in the greenhouse of the Nestlé Plant Science Research Unit (Tours, France). The embryogenic cultures were cultivated following the protocols described previously for *C. arabica*^50^. Briefly, the embryogenic cultures proliferated in 250-ml Erlenmeyer flasks containing M ‘proliferation’ liquid nutritive medium, i.e. a medium supplemented with 0.3 mg/l 2,4-D (2,4-dichlorophenoxyacetic acid) and 1 mg/l BA (6-benzylaminopurine) (Fig. 7a). To stimulate differentiation of early somatic embryos, cultures are “classically” transferred to 250-ml Erlenmeyer flasks containing DIF ‘embryo regeneration’ liquid medium lacking the auxin 2,4-D at a rate of 0.1 g of embryogenic cell clusters per 100 ml of medium for 9 weeks. For miniaturization purposes, 24-well plates (ref. 734–2325, VWR International, Radnor – USA) were chosen as containers for optimum early embryo development. One ml of DIF medium was distributed per well. For automation purposes, the Viaflo Assist automated pipetting platform (Integra Bioscience Corp., Hudson – USA) equipped with a 6-channel pipette (Voyager, Integra Bioscience) was used. Disposable and sterile tips (Cat#4445, 1250 µl), as well as reservoirs (Cat#4322, 100 ml), were also purchased from Integra Bioscience. One g of Arabica cell clusters was first ground with a commercial blender (model: HGB2WT from Waring, Durrington – USA) with a sterilized MC-3 bowl (Waring Products, Durrington – USA) in 100 ml of differentiation medium for 30 sec at 18,000 rpm before filtration on a 700 µm diameter filter, in order to have particle diameters fitting the pipetting tips (Fig. 7b,c). The cell clusters were then inoculated in 24-well plates at an estimated rate of 1 mg per ml per well (Fig. 7d). The 6-channel pipette was programmed in order to have uniform distribution of cell clusters between plates, between rows, and between peripheral and central wells. This programme included a mixing step before pipetting, in order to avoid particle sedimentation. The plates were sealed with Parafilm (M from Bemis, Neenah – USA) and placed in the dark in an incubation shaker (CERTOMAT CT plus, Sartorius Stedim Biotech, Goettingen – Germany) set to 90% RH, 25 °C and 120 rpm for 9 weeks up to the regeneration of torpedo-shaped embryos (Fig. 7e). Uniformity of cluster distribution was assessed at the beginning of culturing, and embryo yield after 9 weeks. This experiment was carried out in 4 replicates with n = 24 wells/replicate.

At the same time, ground and intact cell clusters were cultured in DIF medium under standard *in vitro* conditions (n = 6 flasks/condition/replicate). Erlenmeyer flasks were placed on shakers (120 rpm) at 25 °C in the dark for 9 weeks. The effect of grinding on regeneration capacity was evaluated according to the number of torpedo-shaped embryos obtained per gram of inoculated callus. Total fresh weight was also reported. The experiment was carried out in triplicate.

### Viability test on cell clusters before and after grinding

A viability test was carried out before and after grinding cell clusters. This test consisted of double staining with fluorescein diacetate (FDA) and propidium iodide (PI). In living cells, FDA is degraded by cytosolic esterases allowing it to fluoresce in green (emission at 517 nm) in living cells and PI fluoresces in red (emission at 636 nm) in dead cells. A staining solution was prepared with 5 ml of DIF medium, 8 μl of FDA at 5 g/l (F7378, Sigma Aldrich) and 50 μl of IP at 2 g/l (P4170, Sigma Aldrich). Cell clusters were incubated with 1 ml of staining solution for 10 minutes in the dark before washing 3 times with PBS pH 7.2. Clusters were then suspended in DIF medium and placed between 2 microscope slides for observation.

### Image acquisition and analysis

Images from 24-well plates were automatically acquired using an inverted microscope equipped with a motorized scanning xy stage (Axio Observer D1, Zeiss, Jena – Germany) and a digital camera (Axiocam 503, Zeiss) (Fig. 7f). The software (Zen, Zeiss) was programmed to find the position of each well using auto-focusing (4× objective) and 8 horizontal images by 8 consecutive rows (8 × 8) were taken in order to register each entire well. The resulting collection of 64 reconstructed images per well was later processed using ImageJ freeware (NIH, Maryland – USA) for cell cluster counting and measurement of their diameter, or the total area they occupied in a well.

For viability tests, images of cell clusters were acquired using the same microscope equipped with dichroic filter cubes for the detection of fluorochromes and connected to a UV light source (HXP 120 v, Zeiss).

Images of torpedo-shaped embryos were acquired using a digital camera (Moticam 5+, Motic, Hong-Kong) mounted on a trinocular stereomicroscope (SZH10, Olympus Optical, Hamburg – Germany) and controlled by Motic Images Plus (v.3.0, Motic) software for length measurements.

### Screening of active compounds affecting SE by carrying out HDACi treatments in plates

A full 4 × 4 factorial design of 18 wells replicated 3 times was used to implement the miniaturized bioassay. Four HDACi, Oxamflatin, SAHA, Scriptaid and Trichostastin A (O3139, SML0061, S7817 and T8552, respectively, from Sigma-Aldrich) were tested. Stock solutions of 10 mM were prepared by dissolving the HDACi powders in DMSO, then immediately sterilized by filtration (0.2 μm, Sartorius AG) and diluted in DIF differentiation medium to obtain final concentrations of 0, 0.1, 1 and 10 μM for each HDACi. Ground and filtered Arabica embryogenic clusters were prepared to inoculate 12 24-well plates at an inoculation density of 1 mg/ml of DIF differentiation medium using the Integra pipetting platform (Integra Bioscience Corp., Hudson – USA). For each HDACi, 3 24-well plates were filled with differentiation medium supplemented with 0 (6 wells of row A), 0.1 (wells of row B), 1 (wells of row C) and 10 μM HDACi (wells of row D) respectively (n = 18 wells/HDACi/concentration/replicate). The plates were sealed with Parafilm and placed in an incubation shaker (CERTOMAT CT plus, Sartorius AG) at 90% RH, 120 rpm, 25 °C, in the dark for 24 h. The medium was then gradually replaced with fresh DIF medium by replacing half of the well volume for 6 consecutive times using the pipetting platform (Integra Bioscience Corp.) in order to prevent loss of cell clusters. The plates were placed again in the CERTOMAT for 9 weeks under the same culture conditions. The response to treatment was evaluated according to the number of torpedo-shaped embryos obtained. This experiment was carried out in triplicate.

### TSA re-testing assay under standard *in vitro* culture conditions

TSA at an optimum concentration of 1 µM was re-tested under standard conditions. To that end, Arabica embryogenic cell clusters were inoculated in 24 250-ml Erlenmeyer flasks at a rate of 0.1 g per 100 ml of DIF differentiation medium. Four exposure times to 1 µM TSA were tested (0, 1, 7 and 14 days), at the end of which the medium was replaced with fresh DIF differentiation medium (n = 6 flasks/exposure time/replicate). Flasks were placed on shakers (120 rpm) at 25 °C in the dark for 9 weeks. The response to treatment was evaluated according to the number of torpedo-shaped embryos obtained per gram of inoculated callus. Total fresh weight and mean length of embryos were also recorded. This experiment was carried out in triplicate.

### Plant phenotyping assay post-TSA treatment

Torpedo-shaped embryos from cell clusters treated, or not, with TSA were placed on germination medium in 140 × 21 mm, vented Petri dishes (ref. 391–1500, VWR International) for further development. Dishes were maintained at 25 °C, under a 12/12 photoperiod of artificial light (24 µmol/s, Green Power TLED DR/W/MB 18 W, Philips Lighting, Zurich – Switzerland) delivering 65 µmol.m^−2^.s^−1^ for 2 to 3 months. After cotyledon development, green embryos were transplanted in 145 × 101 × 60 mm plastic boxes (ref: 017002, D. Dutscher S.A., Issy-Les-Moulineaux – France) containing growth medium for shoot and root development under the same conditions as for the Petri dishes. After 4 months, and for each TSA treatment, 40 SE-derived plantlets ready for greenhouse transfer from 3 boxes were assessed as follows: main root and stem lengths, number of pairs of leaves (lp, from 2 to 7) and roots (r, 0–4), number of stems (a, 1 or 2), growth type (orthotropic or plagiotropic), leaf pigmentation (uniform or variegated), leaf colour index (using a green colour chart, Pantone Carlstadt USA) and leaf shape (normal or abnormal).

### Statistical analysis

All analyses were performed in R^51^ using the default functions. First, homogeneity between replicates was verified using a Kruskal-Wallis test (P > 0.05), then normality of data distribution and homogeneity of variances were assessed. If these conditions were verified, the ANOVA test was used to calculate any significant differences (at *P* = 0.05) between the groups (if groups >2), or a t-test was used (if groups = 2). If data did not follow a normal distribution, a Kruskal-Wallis test was used to calculate any significant differences (at *P* = 0.05) between the groups, with a Dunn test as post hoc (dunn.test package).

Based on acclimation data, ANOVA were performed on numerical variables, such as main root and stem lengths, to detect any after-effect of the TSA treatment on plant development. For categorical variables, a multiple component analysis (MCA), performed with Minitab v. 18.1, was used to identify correlations between TSA treatments and phenotypes.

## Supplementary information

Supplementary Information.
