# Supplementary material for: An innovative automated active compound screening system allows high-throughput optimization of somatic embryogenesis in Coffea arabica
Source: Sci Rep. 2020 Jan 21;10:810. doi: 10.1038/s41598-020-57800-6 (PMC6972844; doi:10.1038/s41598-020-57800-6)
Supplement: Supplementary file 1 — Supplementary Information. [file 41598_2020_57800_MOESM1_ESM.pdf]

Nature Scientific Reports

An innovative automated active compound screening system allows high-throughput optimization of somatic embryogenesis in *Coffea arabica*

Rayan Awada[1,2,3]*, Dorothée Verdier[1], Solène Froger[1], Eric Brulard[1], Simone de Faria Maraschin[4], Hervé Etienne[2,3] & David Breton[1]

[1] *Nestlé R&D Center Tours, 101 avenue Gustave Eiffel, 37097 Tours Cedex 2, France*
[2] *CIRAD, UMR IPME, F-34398 Montpellier, France*
[3] *UMR IPME, Université de Montpellier, CIRAD, IRD, F-34398 Montpellier, France*
[4] *Bioalternatives SAS, 1 bis Rue des Plantes, 86160 Gençay, France*
* Correspondence: Rayan Awada, rayan.awada@cirad.fr

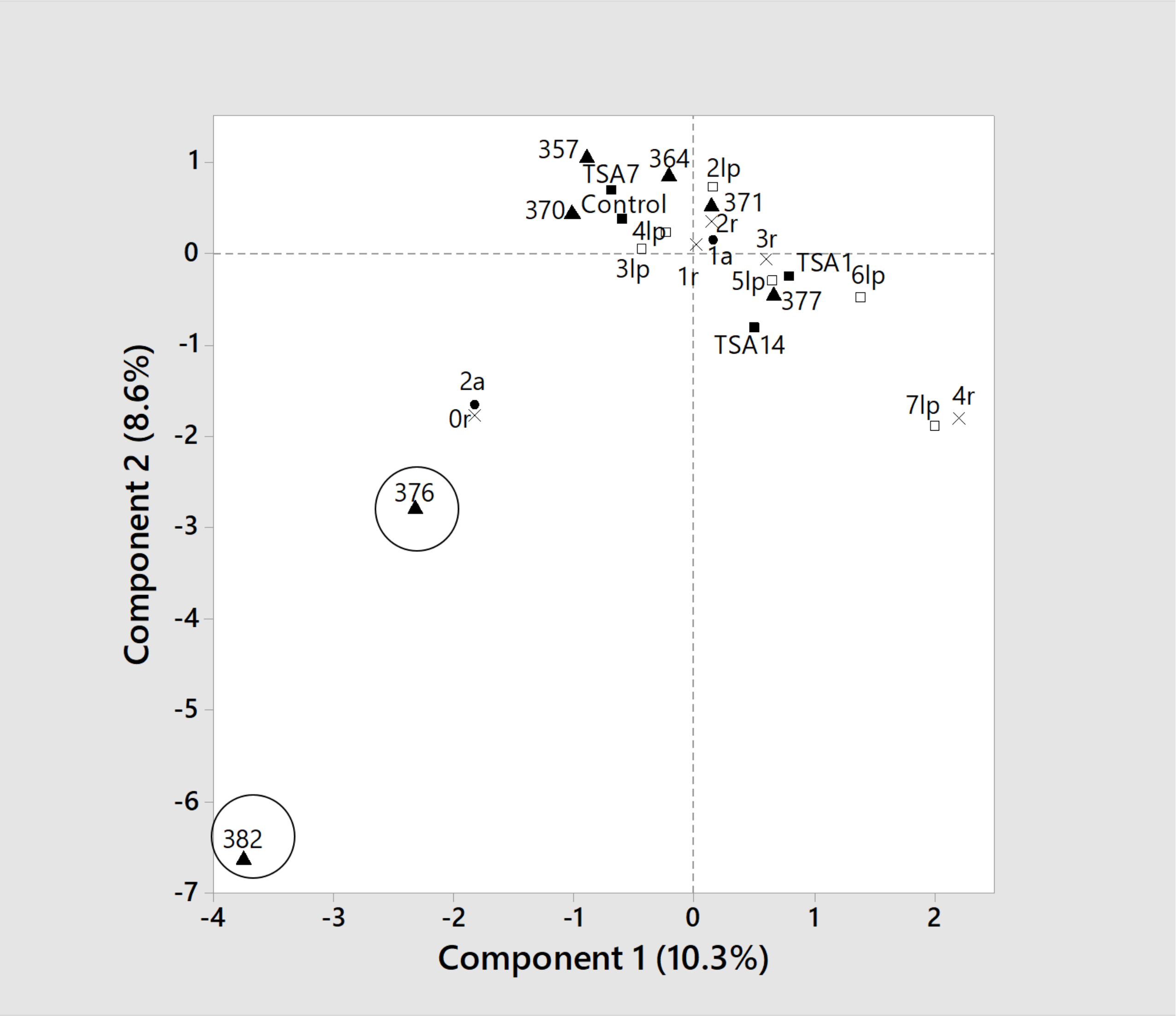

Supplementary data. *In vitro* plant phenotypic characterization following 1 µM TSA treatment during the coffee SE process. A graphic representation of a Multiple Component Analysis (MCA) was performed on plant phenotypic variables. Data from 160 plants were plotted according to the first 2 axes of the MCA. The number of pairs of leaves are represented by white squares, the number of roots by crosses, the number of stems by black spots, the leaf colour index by triangles and the *in vitro* treatment prior to regeneration by black squares. Plotting categorical data according to axes 1 and 2 revealed a strong contribution of 3 atypical plants scored with a single colour index. Therefore, no distinction was detected between TSA treatments. We decided to discard those data and run another MCA (Fig. 6).
